# Mannose enhances the radio-sensitivity of esophageal squamous cell carcinoma with low MPI expression by suppressing glycolysis

**DOI:** 10.1007/s12672-021-00447-0

**Published:** 2022-01-03

**Authors:** Hui Luo, Xiaohui Wang, Yunhan Wang, Qinfu Dan, Hong Ge

**Affiliations:** Department of Radiation Oncology, The Affiliated Cancer Hospital of Zhengzhou University, No. 127 Dongming Road, Zhengzhou, 450008 Henan China

**Keywords:** Mannose, Esophageal squamous cell carcinoma, Mannose phosphate isomerase, Radio-sensitivity, Glycolysis

## Abstract

**Background:**

To investigate the effect of mannose on radio-sensitivity of human esophageal squamous cell carcinoma (ESCC) cell line and its possible mechanism.

**Methods:**

The expression of mannose phosphate isomerase (MPI) in human esophageal cancer cell lines were detected by Western blot. The inhibitory effect of mannose on human esophageal cancer cell lines were observed by MTT assay. Plate clone formation assay was performed to investigate the efficacy of mannose on radio-sensitivity of human esophageal cancer cells. The apoptosis rates of tumor cells treated with mannose and/or radiation therapy was calculated by flow cytometry. Furthermore, we analyzed intracellular metabolites using liquid chromatography mass spectrometry to identify selective sugar metabolites.

**Results:**

MPI expression was various in human esophageal cancer cells. KYSE70 cells was associated with the highest MPI expression whereas KYSE450 cells had the lowest MPI expression level. When administrated with 11.1 mM/L mannose, the same inhibitory effect was observed in both KYSE70 and KYSE450 cell lines. Moreover, the inhibitory effect was significant on KYSE450 cell lines with an increased mannose concentration. The application of 11.1 mM/L mannose could significantly enhance the radio-sensitivity of KYSE450 cell line; and tumor cell apoptosis rate was also increased. However, there was limited efficacy of mannose on the radio-sensitivity and apoptosis rate of KYSE70 cell line. Additionally, intracellular metabolites analyzation revealed that glycolysis could be disturbed by mannose when combined with radiation therapy in esophageal cancer cells.

**Conclusion:**

In esophageal cancer cell lines with low MPI expression, the administration of mannose was associated with enhanced radio-sensitivity.

## Introduction

Mannose was an isomer of glucose, and belonged to one type of monosaccharide. It was naturally existing in a free state. Mannose has been found in a number of berries and vegetables, including cranberries, tomatoes, peaches, cabbage, and green beans. Mannose imported into cells via glucose transporters, and phosphorylated into mannose-6-phosphate by hexokinases [1]. When mannose phosphate isomerase (MPI) exists, mannose-6-phosphate metabolized into fructose-6-phosphate and participated in glycolysis [2]. In addition, with the help of phosphomannomutase, a minor fraction of mannose was isomerized to mannose-1-phosphate and involved in glycosylation, this was essential in the modification and processing of proteins within the endoplasmic reticulum [3].

Previous study reported that mannose suppress lung airway inflammation and experimental type I diabetes by the overexpression of Treg cells; moreover, Treg cell was generated from human naïve CD4^+^ T cells via enhancing TGF-β signaling [4]. Mannose involved in glycosylation during embryonic development, and was a promising supplement in congenital disorder of glycosylation-Ia [5]. It was well established that mannose has anti-inflammation effect by blocking the adherence of bacteria to the urothelium, and was often used for acute or chronic urinary tract infections [6]. In the meantime, several studies showed esophageal cancer patients have significantly higher level of mannose than healthy individuals in serum; mannose could be used as a biomarker of tumor [7, 8]. Recently, Gonzalez et al. reported mannose impaired the invasiveness of cancer cell and enhanced the efficacy of chemotherapy, the results were only observed in tumor cells with low MPI expression [9]. In human blood, physiological mannose level didn’t contribute significantly to cell bioenergetics because it only occupied less than 1% of that of glucose concentration [3]. Therefore, mannose supplement might be a promising method for anti-cancer therapy.

Esophageal cancer was a major global health concern, adenocarcinoma was the widely seen type in Western countries, whereas squamous cell carcinoma was the dominate type in China. Due to the lack of well-established screening system, more than half of the esophageal cancer patients were detected in locally advanced stage. Radiation therapy plays an important role in the treatment of locally advanced malignant tumor, and this anti-cancer strategy was much more effective in esophageal squamous cell carcinoma (ESCC) than esophageal adenocarcinoma [10]. However, the exists of radiation resistance resulted in cancer progression and unfavorable survival [11]. Radiosensitizer were chemical or pharmacologic agents that make tumor cells more vulnerable to radiation therapy [11, 12]. An ideal radiosensitizer was expected to hormone free and harmless to human body, and natural ingredients were preferred.

The purpose of this study was to investigate the value of mannose as a novel radiosensitizer in overcoming radiation resistance and to reveal the underlying mechanism between glycolysis and radio-sensitivity.

## Materials and methods

### Chemicals and reagents

Mannose were obtained from Meilun Biotechnology (Dalian, Cat# 69-65-8). Rosewell Park Memorial Institute (RPMI)-1640 medium (Cat# 31800), Dulbecco’s Modified Eagle Medium (DMEM, Cat# 31600), 3-(4,5-dimethylthiazol-2-yl)-2,5-diphenyltetrazolium bromide (MTT, Cat# M8180), Crystal violet (Cat# C8470), trypsin-EDTA (Cat# T1300), and fetal bovine serum (FBS, Cat# 11011-8611) were purchased from Solarbio Life Sciences. Antibodies against MPI (Cat# sc-393484 AC) was obtained from Santa Cruz Biotechnology. Primary antibody GAPDH (Cat# 97166) and secondary antibodies against mouse (Cat# 7076) were purchased from Cell Signaling Technology.

### Cell lines and cell culture

All cell lines including the esophageal squamous cell carcinoma KYSE30, KYSE70, KYSE140, KYSE150, KYSE410, KYSE450, KYSE510 and the human immortalized normal esophageal epithelial cell line SHEE were obtained from the China-US (Henan) Hormel Cancer Institute stocks and being confirmed to be free of mycoplasma. The esophageal squamous cell carcinoma KYSE30 was grown in high glucose DMEM, whereas KYSE70, KYSE140, KYSE150, KYSE410, KYSE450, and KYSE510 were cultured in RPMI-1640, mixed with 1% streptomycin/penicillin, and 10% FBS (Biological Industries). All cells were maintained in a humidified atmosphere at 37 ℃ and cultured with 5% CO_2_.

### Western blotting

ESCC cells were seeded at 10 cm culture dish (10,000 cells per well) and placed in incubator. The next day, tumor cells were replaced with fresh medium and incubated for 24 to 48 h. After rinsed with ice-cold PBS, cell scrapers were used to collect tumor cells. To obtain total cell lysates, the collected cells were disrupted on ice in NP-40 cell lysis buffer as previously described. The homogenates were centrifuged at 12000*g* for 15 min at 4 ℃ and the protein concentration of supernatant was determined using Bicinchoninic acid (BCA) Protein Assay Kit (Solarbio Life Science, Cat# PC0020). Cell lysates were loaded by SDS-PAGE and blotted onto PVDF membranes. 5% non-fat milk resolved in TBS- Tween 20 (TBST) was used to block the membranes. After an hour, the blocked membranes were incubated overnight with appropriate primary antibodies at 4 ℃. Then the bands were washed with TBST for three times and incubated with a specific HRP-conjugated secondary antibody. At last, the membranes were visualized using Meilunbio^®^ fg super sensitive ECL luminescence reagent (Meilun Biotechnology, Dalian, Cat# MA0186-1).

### Cell culture treatments

Mannose was dissolved in double distilled water and stored at 4 ℃. Tumor cells (200 cells per well) were seeded in a 96-well plate. The next day, the medium were replaced with fresh medium contains different amounts of mannose. During the course of cell proliferate, cancer cell viability was measured by MTT (0.3 mg/mL) assay at 24 h, 48 h, and 72 h as previously described. After the establishment of dose response curve, the inhibitory concentration for each cell lines were calculated.

### RNA interference experiments

The MPI short interfering RNAs (siRNA) were purchased from GenePharma Biotechnology (Shanghai, CN, Cat# A09009). Both KYSE70 and KYSE450 were seeded in 6-well plates in at least triplicate for each experiment, there was without penicillin/streptomycin contained in the medium. When grown to 40–50% confluency, the Lipofectamine RNAiMAX (Thermo Fisher Scientific, Cat# 13778150) was used to transfect cancer cells with the MPI targeting siRNAs, the manufacturer’s instructions were followed during the procedure. Western blot analysis was performed after the successful knockdown of MPI in tumor cells.

### Colony formation assays

Colony formation assays was performed according to standard techniques. Generally, cancer cells (200 cells per well for both KYSE70 and KYSE450) were cultured on 2 mL medium in each well of 6-well plates in triplicate and incubated at 37 ℃ in a 5% CO_2_ incubator. Next day, the culture media were replaced with media containing appropriate concentration of mannose (11.1 mMol/L). After 24 h, tumor cells were irradiated with doses of 0, 2, 4, 6, 8, 10 Gy X-rays using the Varian VitalBeam linear accelerator (Varian Medical Systems, USA). Then cells were further cultured for 12 days until the colonies were optimal. Finally, tumor cells were fixed with methanol and stained with 0.5% crystal violet. Colonies of each well were captured by a microscope and the number of colonies were counted using the Image-Pro Plus software program (version 7.0, Media Cybernetics). To plot the dose survival curves, colonies containing 50 cells or more were analyzed using the classic multi-target single hit model as previously described [11]. The following radiation sensitivity parameters were calculated: survival fraction at 2 Gy (SF2), mean lethal dose (D0), and quasithreshold dose (Dq).

### Flow cytometry

Tumor cells were seeded at a concentration of 300,000 cells per well in a 6-well plate overnight. The next morning, cells were treated with mannose (11.1 mMol/L) or radiation therapy (4 Gy). Then cells were incubated at 37 ℃ in a 5% CO_2_ incubator. After 48 h, these cells were collected, both annexin V-FITC and propidium iodide were added for cell death assay. A BD FACSCalibur Flow Cytometer (BD Biosciences) was used for the analysis.

### Analysis of intracellular metabolites

Cancer cells (100,000 cells per well) were cultured in 6-well plates. The medium was replaced next day, and incubated for 24 h at 37 ℃. Then, these cells were treated with mannose (11.1 mMol/L) and/or radiation therapy (4 Gy) and cultured for 6 h. Next, intracellular metabolites were extracted by the addition of extraction solvent and centrifuged at 12,000 revolutions per minute for 15 min at 4 ℃. Then, the supernatants were analyzed using liquid chromatography mass spectrometry (6460 Triple Quad LC/MS, Agilent Technologies) to identify selective sugar metabolites.

### Statistical analysis

All presented data were analyzed using SPSS 20.0 (IBM Software Group) and Prism 7 (GraphPad Software). Each experiment was carried out three times. The quantitative data were calculated as mean ± standard deviation (SD). Statistically significant differences were determined using the Student’s t-test to compare data between two groups, or one-way ANOVA and the Bonferroni correction to compare data among three or four groups. Statistical significances were set as a two-tailed *p* < 0.05 (*p < 0.05, **p < 0.01, ***p < 0.001).

## Results

### Treatment with mannose attenuated tumor cell proliferation

**Fig. 1:**
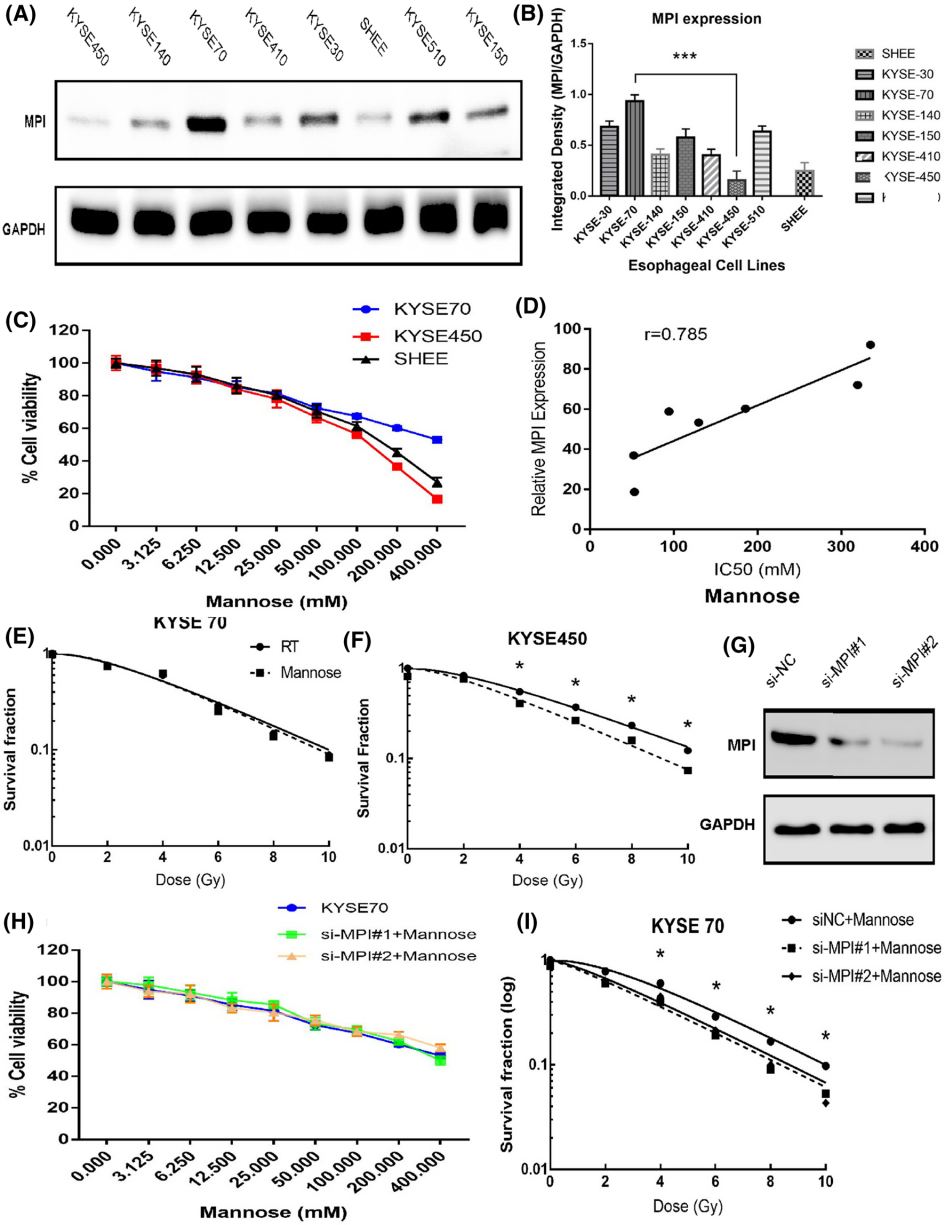
(1) MPI protein expression in different ESCC cell lines and mannose inhibits the growth of ESCC cells (**A**–**D**). **A** The expression of MPI in ESCC cells and normal esophageal epithelial cells (SHEE). **B** Quantification of MPI expression from (**A**). **C** Dose-survival curves of mannose on KYSE70 cells, KYSE450 cells and SHEE cells were estimated by MTT assay at 24 h. **D** Correlation of IC50 and MPI expression. r is the Pearson correlation coefficient. (2) Mannose enhances the sensitivity of radiation therapy in ESCC cells with low MPI expression. **E** Colony formation assay was performed in KYSE70 cells following the DMSO (vehicle), Mannose (11.1 mM), RT (0, 2, 4, 6, 8, and 10 Gy), or RT/Mannose treatment. **F** Colony formation assay was performed in KYSE450 cells following the DMSO (vehicle), Mannose (11.1 mM), RT (0, 2, 4, 6, 8, and 10 Gy), or RT/Mannose treatment. **G** Western bolt of KYSE70 cells infected with siRNAs targeting MPI. **H** Dose response curves were generated in KYSE70 cells transfected with siRNAs targeting MPI and KYSE70 cells in the absence or presence of mannose for 24 h. **I** Colony formation assay was performed in KYSE70 cells infected with siRNAs targeting MPI and mannose. *ESCC* esophageal squamous cell carcinoma, *RT* radiation therapy, *siNC* negative control siRNA, *MPI* mannose phosphate isomerase, *IC50* the half maximal inhibitory concentration. Data are means ± SD (n = 3), n.s., not significant, p > 0.05; *p < 0.05; **p < 0.001; ***p < 0.0001

Since MPI has been proved to play an essential role in mannose metabolism [9], we measured the intracellular MPI levels from seven different ESCC cells and one normal esophageal epithelial cell (SHEE) using western bolt analysis. Among tumor cells, KYS450 was associated with the lowest expression of MPI, whereas KYSE70 was correlated with the highest expression of MPI (Fig. 1A, B); meanwhile, SHEE cell line was also associated with low MPI expression. Next, cell proliferation assay was performed to determine the inhibitory effect of mannose on ESCC cells (KYSE70 and KYSE450) and normal esophageal epithelial cell SHEE. Our results suggested that mannose inhibits cell proliferation in a dose-dependent manner, especially in cells with low MPI expression (KYSE450 and SHEE) (Fig. 1C).

### MPI levels were correlated with mannose sensitivity

Then, we evaluated the correlation of MPI expression and mannose sensitivity in various cell lines. Our findings revealed that mannose has the same inhibitory effect of 13.3% on KYSE70 cell lines, KYSE450 cell lines, and SHEE cell lines at a concentration of 11.1 mMol/L. Moreover, KYSE450 was much more sensitive to mannose than KYSE 70. The IC_50_ values of mannose was positively correlated with relative MPI expression in ESCC cell lines (Fig. 1D). These results suggested that MPI involved in the regulation of mannose metabolism.

### The synergistic effect of radiation therapy and mannose

Overcoming radiation resistance was a great challenge in cancer therapy. To evaluate the synergistic effect of radiation therapy and mannose, colony formation assays were performed in ESCC cells (KYSE450 and KYSE70). Because 11.1 mMol/L mannose was associated with invisible signs of toxicity, both KYSE70 and KYSE450 cells were treated with this concentration of mannose. The combination of mannose and radiation therapy showed a significant reduced survival fraction in KYSE450 cells when compared with radiation therapy alone. However, the radiation sensitization effect was not observed in KYSE70 cells (Fig. 1E, F). These findings suggested that mannose may act as a novel radiosensitizer, and this effect was MPI dependent. Similar results were observed in other ESCC cell lines.

Furthermore, to confirm the role of MPI inhibition in regulating radiation sensitization, siRNA inference experiments were conducted. MPI knockdown only did not affect tumor cell viability (Fig. 1G, H). When combined with mannose, MPI knockdown sensitizes KYSE70 cells to radiation therapy (Fig. 1I). Collectively, these outcomes suggested that MPI was the key factor in regulating the radiation sensitivity of mannose.

### Mannose induced cell death through the apoptotic pathway

**Fig. 2:**
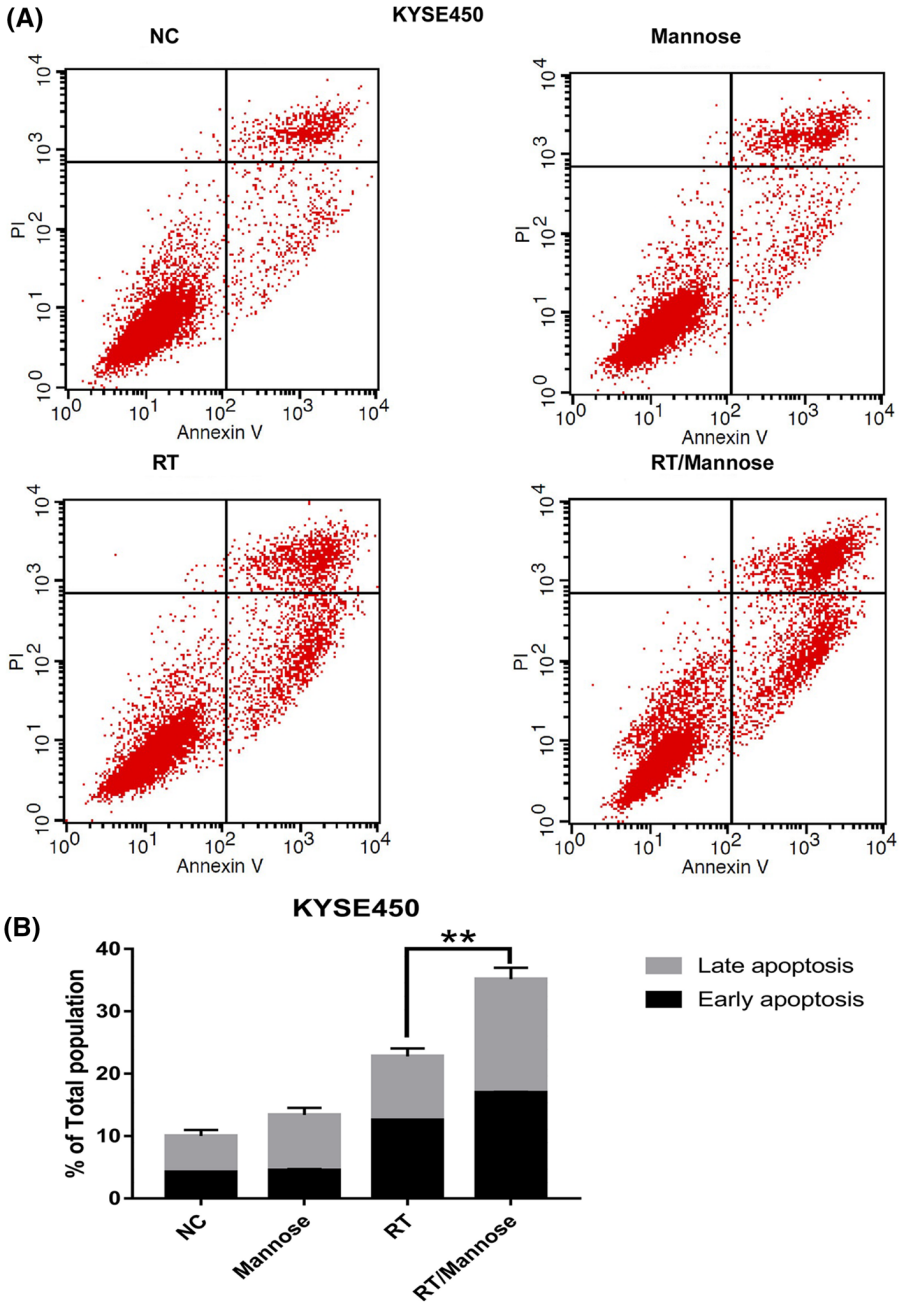
Mannose enhanced the efficacy of radiation therapy induced apoptosis in KYSE450 cells. **A** Annexin-V/PI staining was performed following 24 h of DMSO (vehicle), Mannose (11.1 mM), RT (6 Gy), or RT/Mannose treatment in KYSE450 cells. **B** Quantification of apoptosis from (**A**). *NC* normal control, *RT* radiation therapy. Data are means ± SD (n = 3), n.s., not significant, p > 0.05; *p < 0.05; **p < 0.001; ***p < 0.0001

**Fig. 3:**
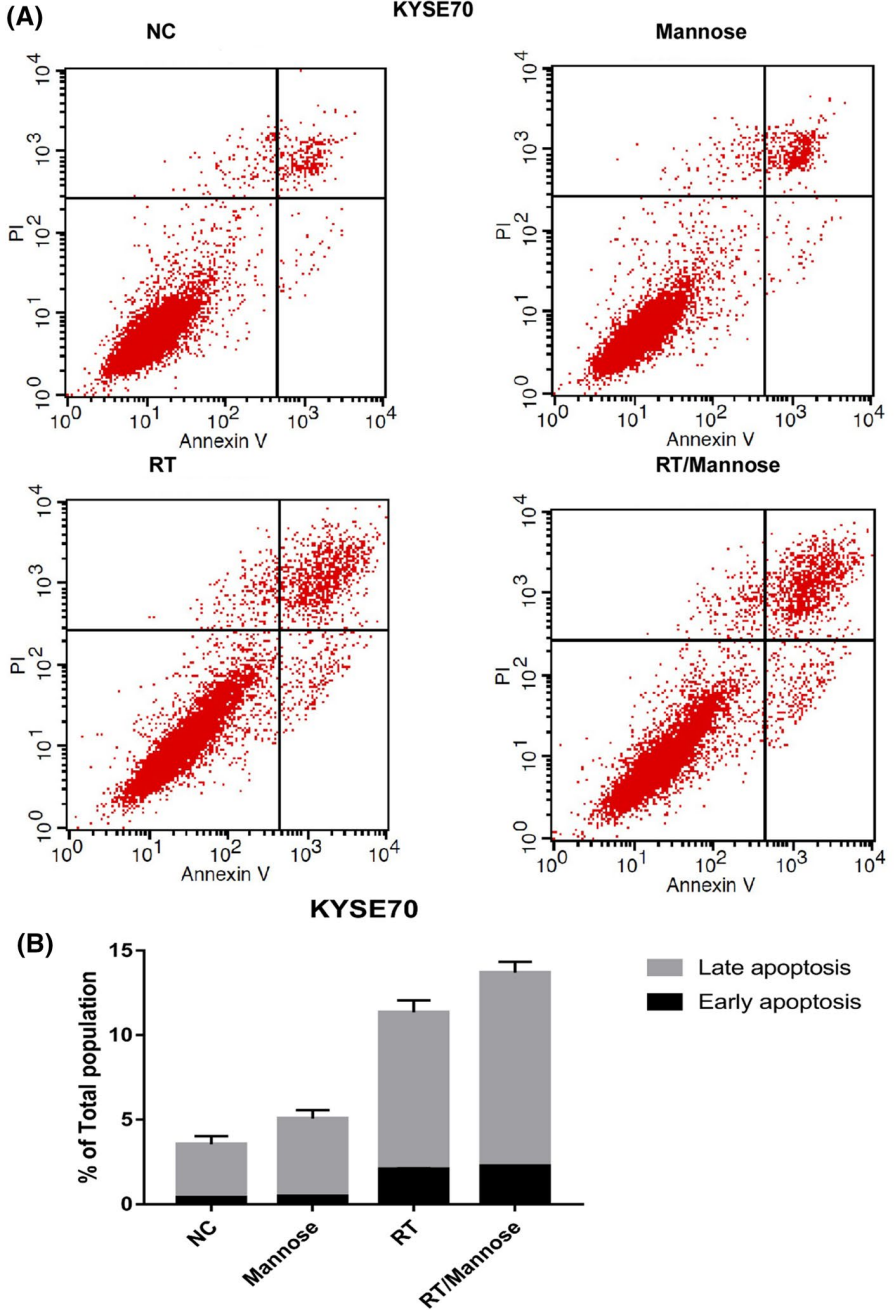
Mannose failed to improve the efficacy of radiation therapy induced apoptosis in KYSE70 cells. **A** Annexin-V/PI staining was performed following 24 h of DMSO (vehicle), Mannose (11.1 mM), RT (6 Gy), or RT/Mannose treatment in KYSE70 cells. **B** Quantification of apoptosis from (**A**). *NC* normal control, *RT*: radiation therapy. Data are means ± SD (n = 3), n.s., not significant, p > 0.05; *p < 0.05; **p < 0.001; ***p < 0.0001

On the basis of the above data, mannose was effective in enhancing the radiation sensitivity of ESCC cell lines with low MPI expression, we further investigated whether this combination strategy triggers cell death. The quantitation of apoptosis was determined by annexin V binding, Propidium iodide uptake, and flow cytometry. Generally, mannose has limited effects in inducing ESCC cells apoptosis, radiation therapy alone was associated with higher apoptosis rate than mannose alone. In KYSE450 cells, the results also showed a significant higher late apoptosis rates in the combined group when compared with radiation therapy alone (Fig. 2A, B). However, there was insignificant increasement of apoptosis rate of KYSE70 cells treated with radiation therapy and mannose (Fig. 3A, B). Next, we explored whether MPI knockdown was able to enhance the combined strategy in inducing tumor cell apoptosis. Interestingly, knock down of MPI only was slightly toxicity in KYSE70 cells. Mannose was efficient in improving radiation induced apoptosis in tumor cells without MPI expression.

**Fig. 4:**
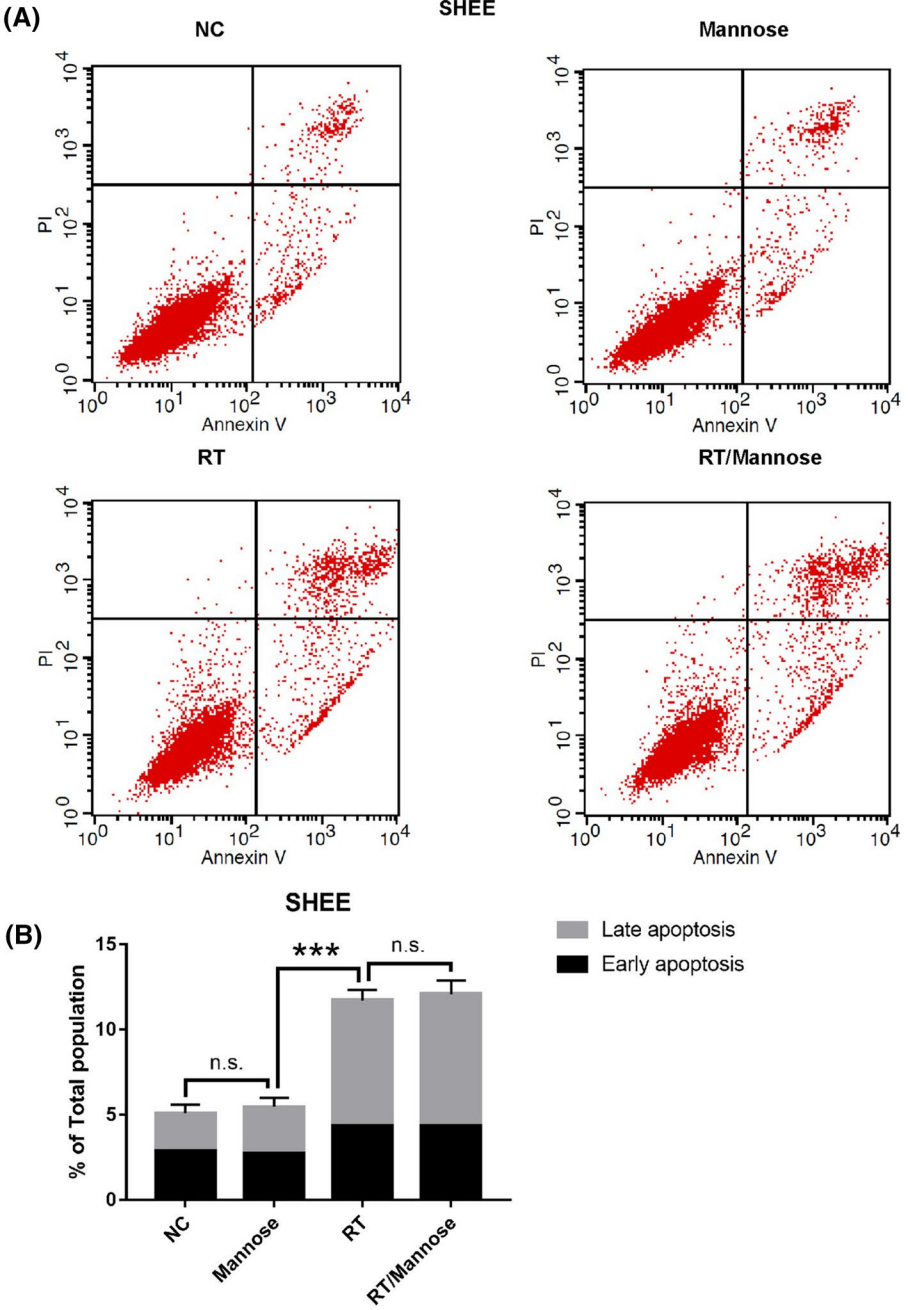
Mannose failed to improve the efficacy of radiation therapy induced apoptosis in SHEE cells. **A** Annexin-V/PI staining was performed following 24 h of DMSO (vehicle), Mannose (11.1 mM), RT (6 Gy), or RT/Mannose treatment in SHEE cells. **B** Quantification of apoptosis from (**A**). *NC* normal control, *RT* radiation therapy. Data are means ± SD (n = 3), n.s., not significant, p > 0.05; *p < 0.05; **p < 0.001; ***p < 0.0001

In addition, the toxicity of mannose in normal esophageal epithelial cells has been evaluated. We found that mannose was slightly toxic in normal cells, and radiation therapy combined with mannose was insignificant in triggering tumor cell death (Fig. 4A, B).

Taken together, the above results suggested that mannose enhanced radiation induced apoptosis, and this effect was only observed in tumor cells with low MPI expression.

### The combination of mannose and radiation therapy resulted in glucose metabolism disorder

**Fig. 5:**
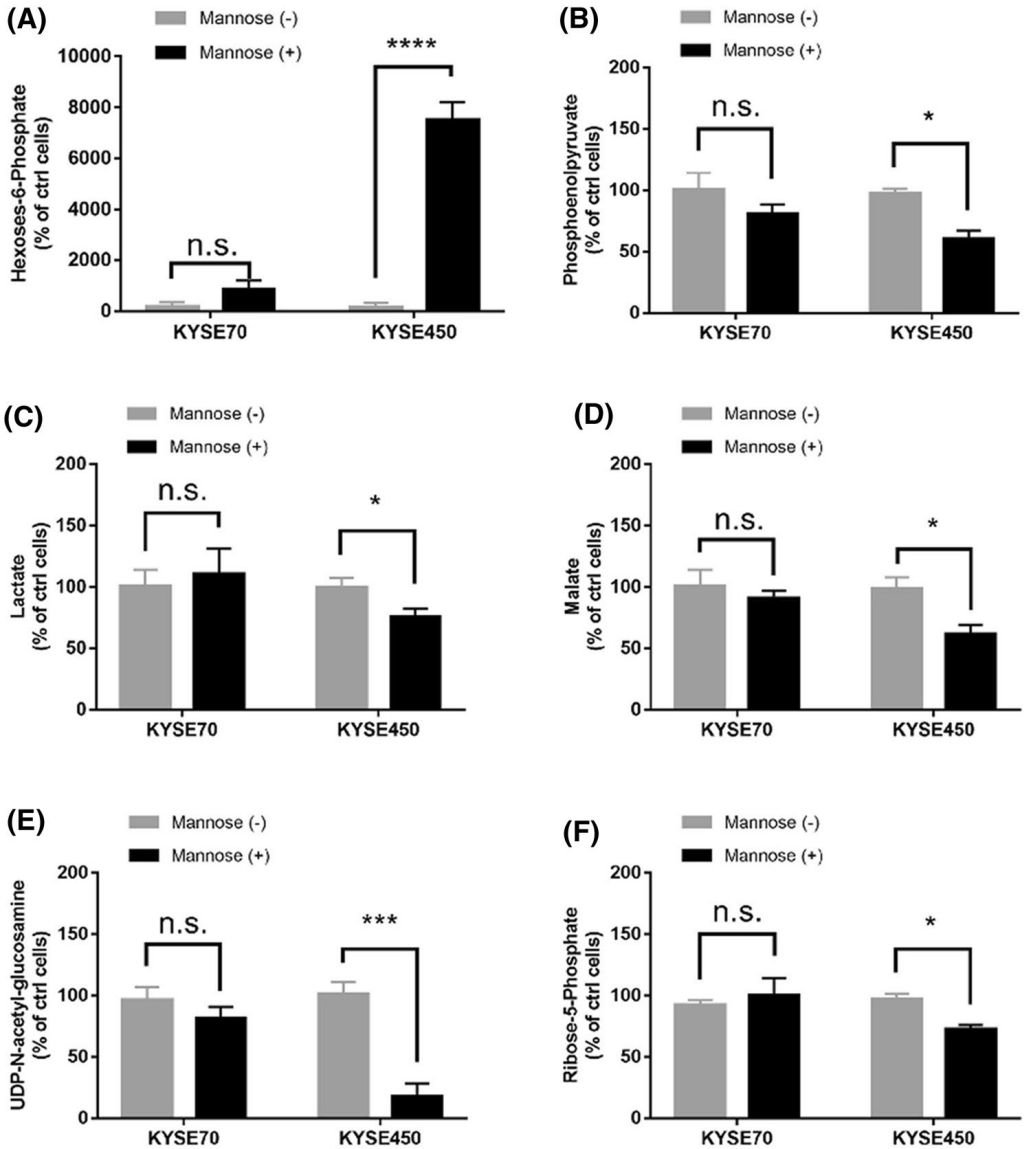
Mannose combined with radiation therapy resulted in glucose metabolism disorders. KYSE70 cells and KYSE 450 cells were treated with vehicle, or Mannose (11.1 mM), and the changes in cellular metabolism were analyzed by LC/MS. **A** Relative levels of hexoses-6-phosphate, **B** Relative levels of phosphoenolpyruvate. **C** Relative levels of lactate. **D** Relative levels of malate. **E** Relative levels of UDP-*N*-acetyl-glucosamine. **F** Relative levels of Ribose-5-phosphate. *LC/MS* liquid chromatography/mass spectrometry. Data are means ± SD (n = 3), n.s., not significant, p > 0.05; *p < 0.05; **p < 0.001; ***p < 0.0001

**Fig. 6:**
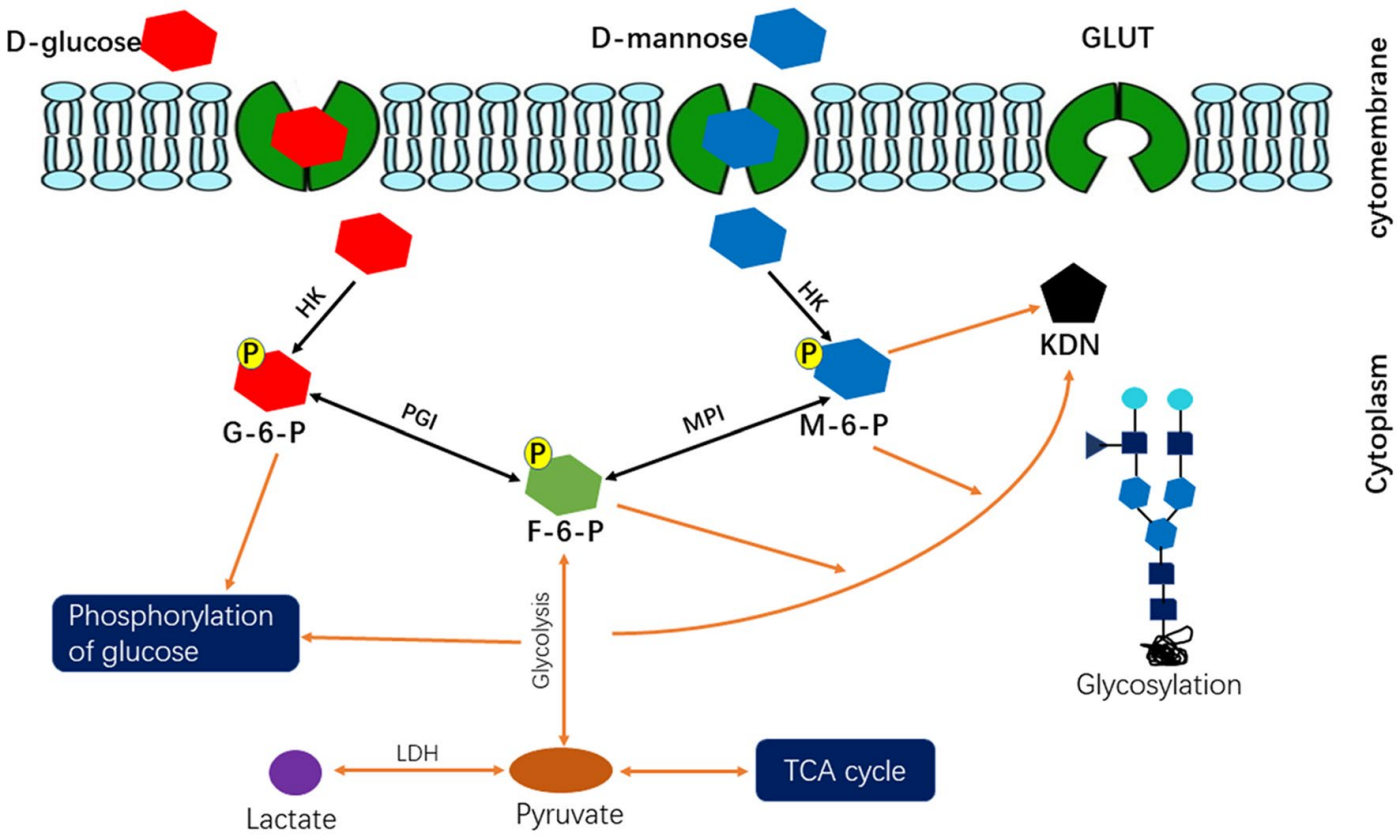
Mechanisms of mannose enhances the efficacy of RT in human ESCC cell lines with low MPI expression. *ESCC* esophageal squamous cell carcinoma, *F-6-P* fructose 6-phosphate, *G-6-P* glucose 6-phosphate, *GLUT* glucose transporter glucose transporter, *HK* hexokinase, *KDN* deaminoneuraminic acid, *LDH* lactate dehydrogenase, *M-6-P* mannose 6-phosphate, *MPI* mannose phosphate isomerase, *RT* radiation therapy, *PGI* phos-phoglucose isomerase, *TCA cycle* tricarboxylic acid cycle

Glucose metabolism played an active role in tumor cells proliferation [13]. Previous study revealed that glucose metabolization was involved in tumor cells radiation resistance [14]. Mannose could be phosphorylated to mannose-6-phosphate by hexokinase, MPI catalyzed the interconversion of fructose-6-phosphate and mannose-6-phosphate and this was crucial for most glycosylation reactions [15]. To address the role of mannose in glucose metabolism, we measured the levels of hexoses-6-phosphate, phosphoenolpyruvate, lactate, malate, UDP-*N*-acety-glucosamine, and ribose-5phosphate. We observed that mannose interfered with glucose metabolism in KYSE450 cells by inhibiting glycolysis, oxidation of pyruvate, citric acid cycle, glycosylation, and pentose phosphate pathway. Interestingly, disturbed glucose metabolism was not detected in KYSE70 cells treated with mannose (Fig. 5A–F). These findings suggested that glucose metabolization was interrupted by mannose in tumor cells with low MPI expression. Under the condition of less energy supply in tumor cells with high expression of MPI, mannose-6-phosphate was converted to mannose-6-phosphate and contributes to glucose metabolism. Therefore, radiation induced glucose metabolism disorder was repaired and resulted in radiation resistance (Fig. 6).

Overall, radiation therapy combined with mannose causing severe glucose metabolization disorders, this was sufficient to choke off the energy supply of cancer cells and to trigger apoptosis. By the way, the effects of mannose on glucose metabolism were only observed in tumor cells with low MPI expression.

## Discussion

Tumors cells exhibit increased uptake of glucose to survival themselves. Generally, glucose enter the cells through the glucose transporter 2 (GLUT2). With the help of enzyme hexokinase and glucose phosphate isomerase, glucose was phosphorylated and converted to fructose-6-phosphate. Similarly, with the utilizes of various enzymes, energy was generated mainly through the metabolic process of glycolysis. There were several pathways involved in glucose metabolism, and anaerobic glycolysis played a key role in cancerogenesis and tumor progression [16]. Even in the presence of an adequate oxygen supply, most tumor cells rely disproportionately on glycolysis for energy supply [17]. Moreover, the compounds produced through aberrantly activated glycolysis in tumor cells can enter into a variety of metabolic pathways, such as pentose phosphate pathway and oxidative phosphorylation pathway [18]. Previous studies revealed that the dysregulated expression of multiple glycolysis-related enzymes during carcinogenesis [16, 19, 20]. Therefore, enzymes involved in glycolytic metabolism were become promising targets, inhibit the glycolytic pathway would decreasing the energy supply, reducing cell proliferation, stimulating apoptosis, and resulted in a reduction of tumor growth.

Extracellular mannose entered to the cytosol of the cells via GLUT2, the same transport as glucose; it can be converted to mannose-6-phosphate in cells with the help of hexokinase, and this undermines the further metabolism of glucose in glycolysis, glycan synthesis, the pentose phosphate pathway and the tricarboxylic acid cycle, both directly and indirectly [21]. Glucose metabolism disorder resulted in energy depletion, which was effectively in suppressing tumor growth [2, 9]. DeRossi et al. reported that mannose-6-phosphate inhibited the activity of hexokinase, glucose phosphate isomerase, and glucose-6-phosphate dehydrogenase, this impaired the further metabolism of glucose and induced a decrease in cell proliferation [2]. The present study showed that mannose inhibited the proliferation of esophageal cancer cells, and the cytotoxicity of mannose was in a dose and time dependent manner. MPI catalyzed the interconversion of mannose-6-phosphate and fructose 6 phosphate, and played an essential role in glucose metabolism [22]. MPI also has a decisive role in tumor suppression, knockdown of MPI resulted in a significant increased cytotoxicity of mannose on tumor cells [9]. In the current analysis, we also found that KYSE70 cells were associated with the highest expression of MPI whereas KYSE450 was correlated with the lowest expression of MPI; when treated with the same concentration of mannose, cell proliferation was significantly decreased in KYSE450 cell lines when compared with the KYSE70 cell line. The results also indicated that the antitumor efficacy of mannose was negatively correlated with MPI expression.

During radiation therapy, tumor cells developed hypoxia due to insufficient blood supply, this resulted in upregulated and elevated cellular activity of glycolytic enzymes and an increased production of lactate; cellular lactate was an important reactive oxygen species, and played a crucial role in decreasing radiation sensitivity [23, 24]. Leung et al. demonstrated that down-regulated hypoxia-inducible factors enhanced tumor response to radiation therapy via reducing lactate production and impairing glycolysis [25]. In the present study, we observed mannose contributes to the radiation sensitivity of KYSE450 cell lines; the possible mechanism was that uptake of mannose by glucose transporters drives the accumulation of mannose-6-phosphate, which in turn impairs tumor glycolysis and enhances the efficacy of radiation therapy in cancer patients with low MPI expression.

Mannose was a natural bioactive monosaccharide, and has been utilized as dietary supplement influencing glyconutrient in clinical practice. Under physiological conditions, mannose accounted for less than 2% of the concentration of blood glucose and was mainly involved in protein glycosylation in vivo [5]. Radiation therapy has a vital role in esophageal cancer. The current study demonstrated that mannose was able to inhibit esophageal cancer cell proliferation; in tumor cells with low MPI expression, mannose combined with radiation therapy inhibited cell viability, induced glucose metabolism disorder and resulted in cancer cell death. Accordingly, radiation sensitivity was enhanced. The present study will provide novel targets for improving tumor response to radiation therapy.

In conclusion, mannose was widely used in pharmaceutical and food industries, and its safety and tolerability have been tested. Therefore, in esophageal squamous cell carcinoma patients with low MPI expression, mannose could be used as a radiation sensitizer, the application of mannose was simple and easy, and have broad application prospects.

## Data Availability

The data used to support the findings of this study are available from the corresponding author upon request.

## Abbreviations

BCA: Bicinchoninic acid
DMEM: Dulbecco’s Modified Eagle Medium
ESCC: Esophageal squamous cell carcinoma
FBS: Fetal bovine serum
F-6-P: Fructose 6-phosphate
G-6-P: Glucose 6-phosphate
GLUT: Glucose transporter glucose transporter
HK: Hexokinase
KDN: Deaminoneuraminic acid
LDH: Lactate dehydrogenase
M-6-P: Mannose 6-phosphate
MPI: Mannose phosphate isomerase
MTT: 3-(4,5-Dimethylthiazol-2-Yl)-2,5-diphenyltetrazolium bromide
RT: Radiation therapy
PGI: Phos-phoglucose isomerase
SD: Standard deviation
TCA cycle: Tricarboxylic acid cycle
